# Supplementary material for: Achieving three-dimensional lithium sulfide growth in lithium-sulfur batteries using high-donor-number anions
Source: Nat Commun. 2019 Jan 14;10:188. doi: 10.1038/s41467-018-07975-4 (PMC6331553; doi:10.1038/s41467-018-07975-4)
Supplement: Supplementary file 1 — Supplementary Information [file 41467_2018_7975_MOESM1_ESM.pdf]

Supplementary Information for

Achieving three-dimensional lithium sulfide growth in lithium-sulfur batteries using high-donor-number anions

Chu et al.

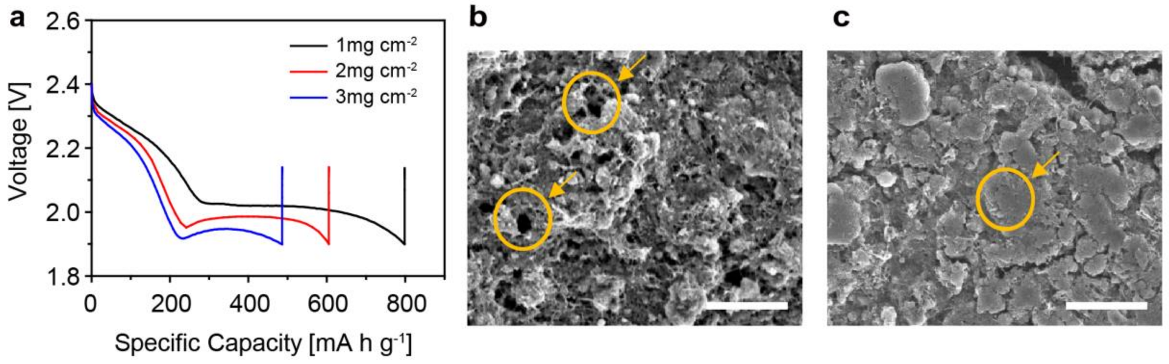

Supplementary Figure 1 | Voltage profiles and Scanning Electron Microscopy images of conventional Ketjenblack/sulfur composite electrodes (**a**) The capacity driven from the lower plateau reaction was decreased with increasing the sulfur loading of the electrode. It is presumed to originate from random deposition of insulating Li_2S on the electrode top surface, then the insulating layer causes faster electrode passivation before utilizing all of the loaded active mass. Scanning Electron Microscopy (SEM) images of (**b**) before cycling and (**c**) after discharge the 3 mg cm^{-2} Ketjenblack/sulfur (KB/S) electrode. The pores, which contribute as ion supply channels through the electrode, were almost clogged by the random and lateral deposition of Li_2S. The electrolytes consist of 1 M lithium bis(trifluromethanesulphonyl) imide (LiTFSI) / 0.2 M lithium nitrate ($LiNO_3$) / 1,3-dioxolane (DOL): 1,2-dimethoxyethane (DME) (1:1) Discharge C rate: 1 C, Scale bars, 20 μm (**b-c**)

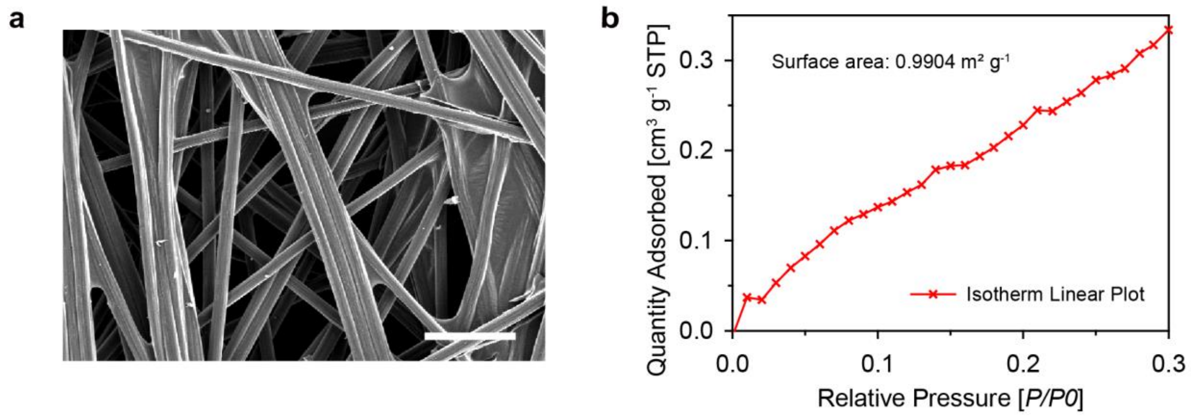

Supplementary Figure 2 | Characterization of a pristine carbon paper electrode (**a**) A Scanning Electron Microscopy (SEM) image of a pristine carbon paper (CP) electrode shows tens of micro-meter-scaled macro pores within fibrous carbon frameworks. Scale bar, 50 μm (**b**) The Brunauer–Emmett–Teller (BET) isotherm of the CP electrode. BET surface area of the CP electrode was measured as a value of 0.9904 m^2 g^{-1}.

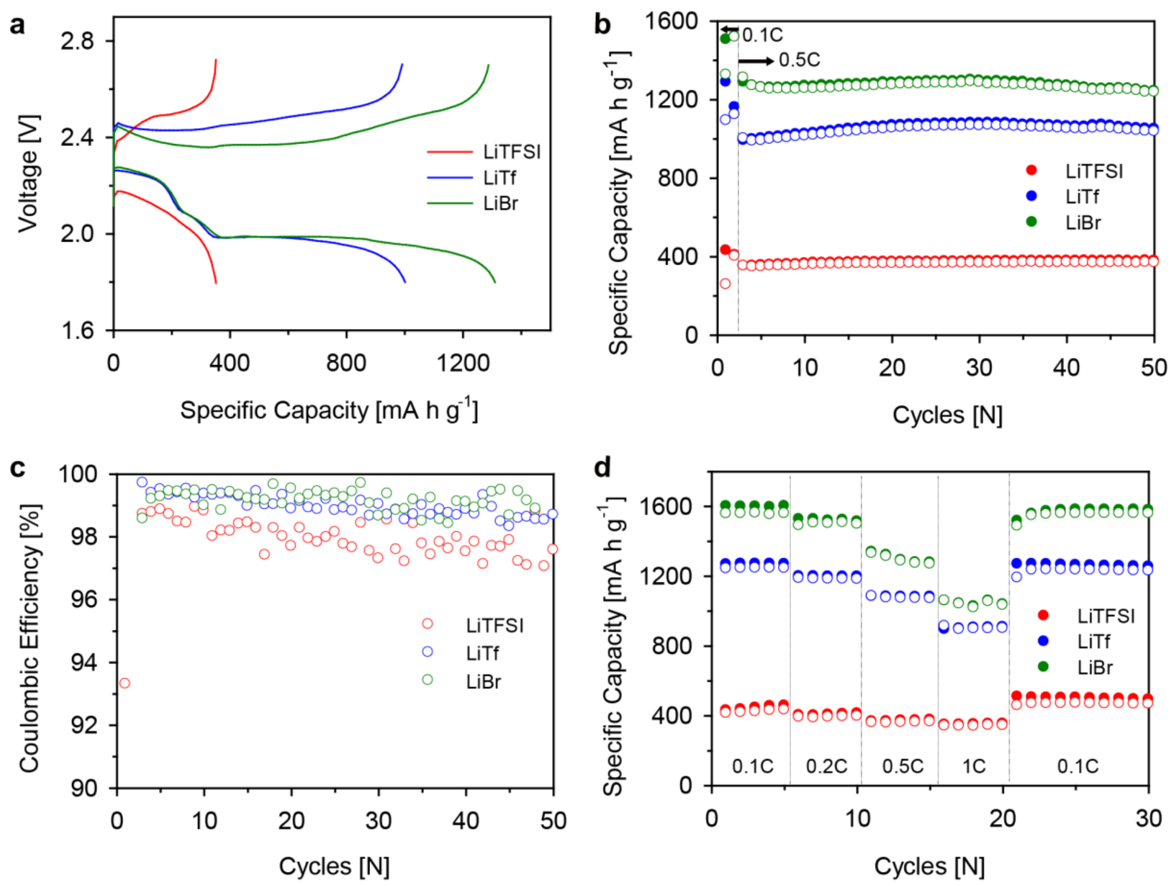

Supplementary Figure 3 | Electrochemical performances at higher current densities with the LiTFSI (red), LiTf (blue), and LiBr (green) electrolytes (**a**) Charge and discharge profiles at the first cycle (0.5 C), (**b**) comparison of the charge (closed circle) and discharge (open circle) capacities during the cycling at 0.5 C, (**c**) coulombic efficiencies for 50 charge/discharge cycles at 0.5 C, and (**d**) rate capability test with increasing current density from 0.1 C to 1 C and recovering to 0.1 C. The electrolytes consist of 0.2 M lithium polysulfide (LiPS, Li_2S_8 based) with 1 M Li salts (LiX, X= TFSI, Tf, or Br) / 0.2 M lithium nitrate ($LiNO_3$) / 1,3-dioxolane (DOL): 1,2-dimethoxyethane (DME) (1:1), Theoretical areal capacities: 1.68 mA h cm^{-2}

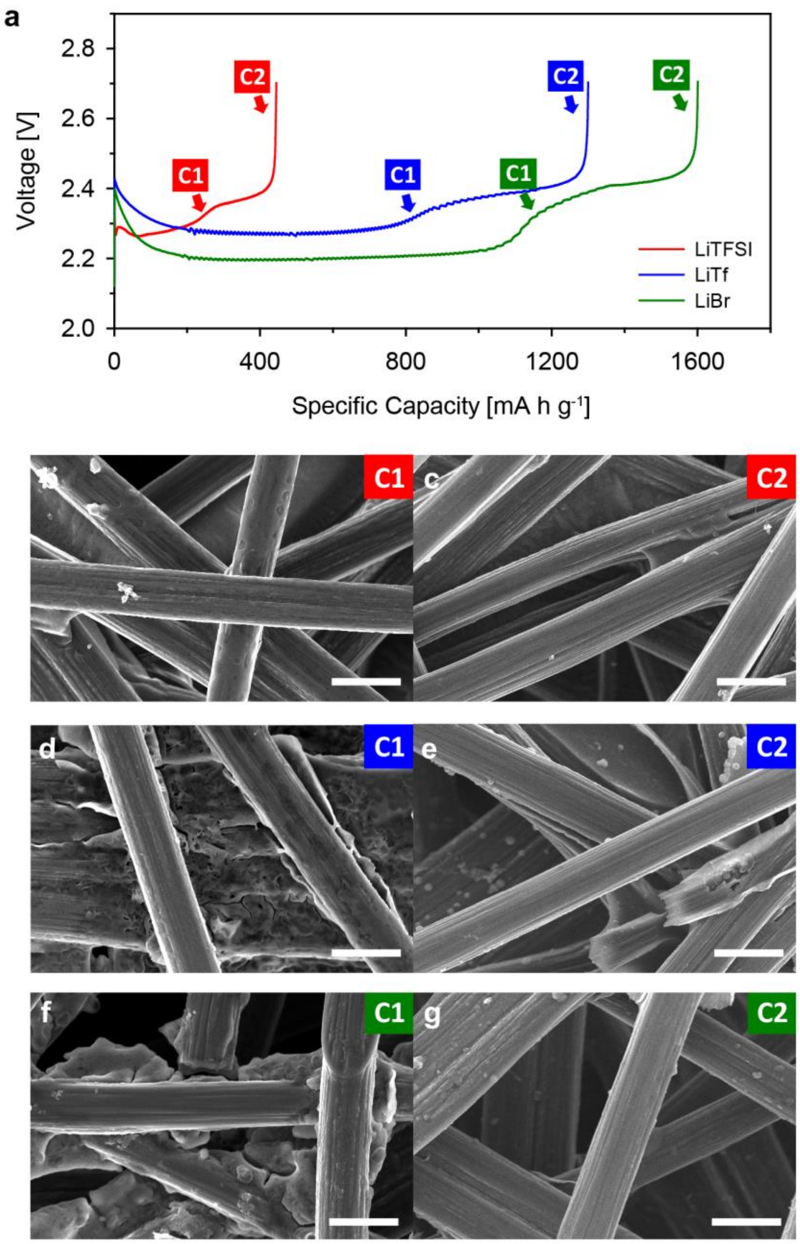

Supplementary Figure 4 | Scanning Electron Microscopy images of the charged cathodes at different states of charge with the LiTFSI, LiTf, and LiBr electrolytes (**a**) The succeeding charge profiles at 0.05 C after the first discharge in Fig. 2. The Scanning Electron Microscopy (SEM) images for (**b-c**) LiTFSI, (**d-e**) LiTf, (**f-g**) LiBr were taken by charging the separate cells until the two different states of charge (SOC) marked as C1 (2.3 V) and C2 (fully charged). Scale bars, 10 μm

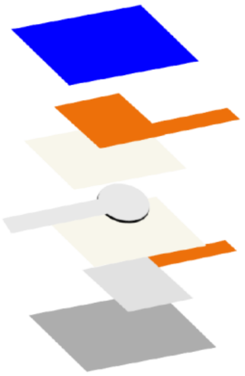

Supplementary Figure 5 | The detailed configuration of three-electrode pouch type cell for Electrochemical Impedance Spectroscopy analyses For distinguishing the impedance contribution from the cathode and anode, a lithium (Li) metal reference electrode was inserted at the specified position in the figure, where can avoid the influence of the electric field during the Electrochemical Impedance Spectroscopy (EIS) analysis. Stainless steel was selected as a current collector material of the counter and the reference electrodes to minimize the side reaction with lithium polysulfide (LiPS) species[1]. Aluminum foil was employed as a cathode current collector.

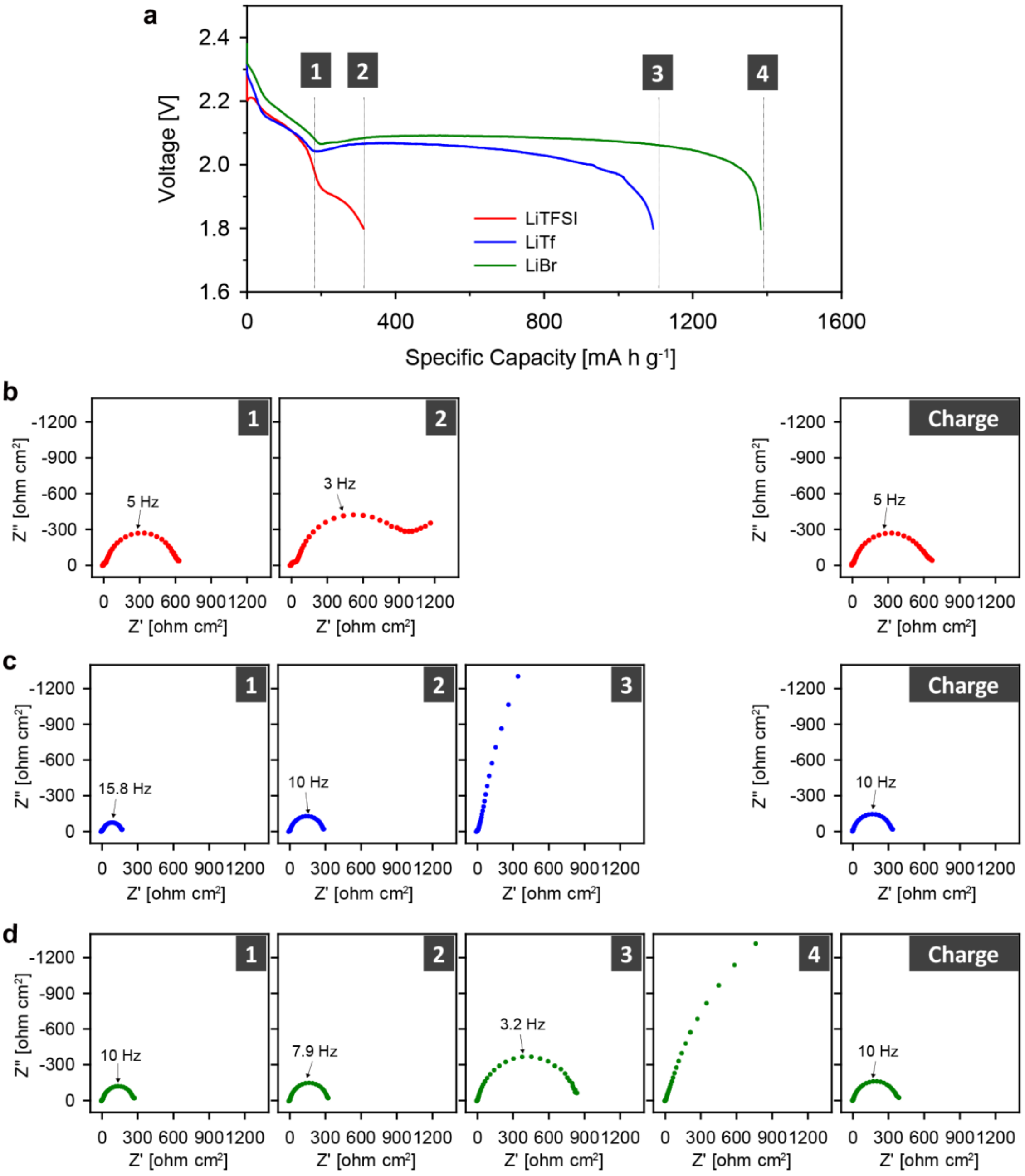

Supplementary Figure 6 | Impedance measurements of the carbon paper cathodes discharging with the LiTFSI (red), LiTf (blue) and LiBr (green) catholytes (**a**) The first discharge profiles of three-electrode pouch-type cells with the different salt anions at 0.05 C rate. The impedance values of the cathodes were separately obtained at each corresponding states of discharge (SOD), marked as 1-4, and after complete re-charge with 1 M (**b**) LiTFSI, (**c**) LiTf, and (**d**) LiBr based 1,3-dioxolane (DOL): 1,2-dimethoxyethane (DME) (1:1) / 0.2 M lithium nitrate ($LiNO_3$) / 0.2 M lithium polysulfide (LiPS) electrolytes

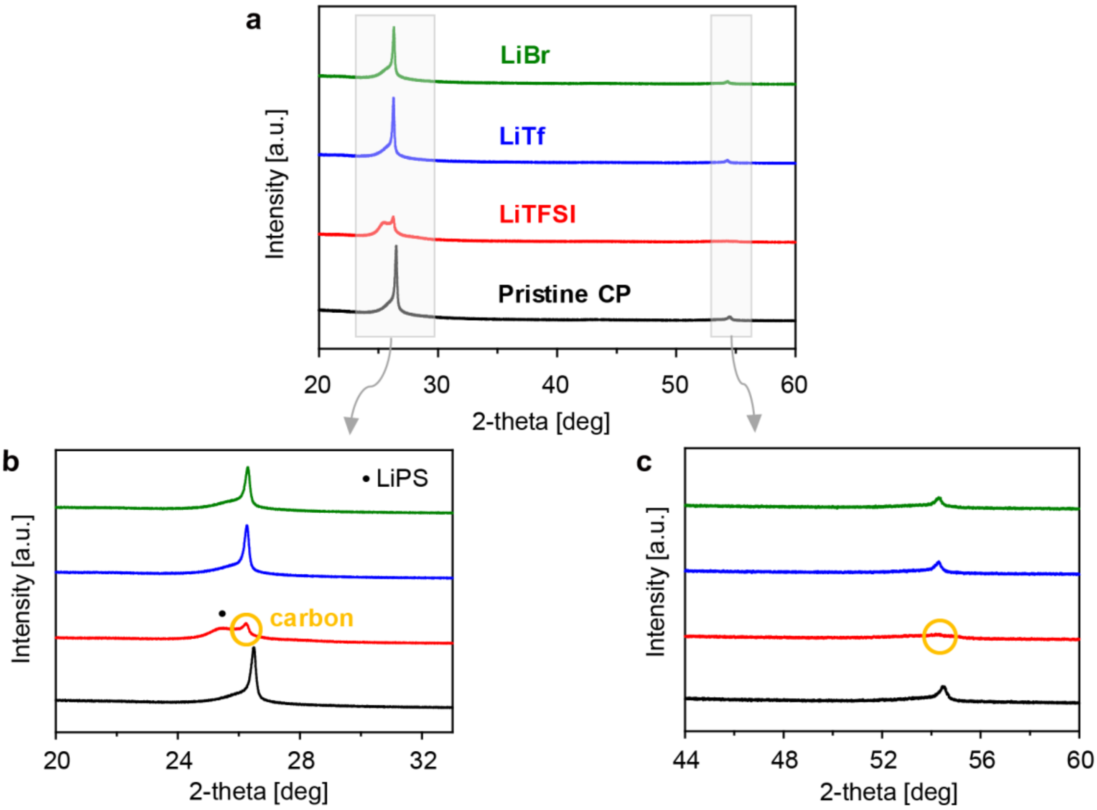

Supplementary Figure 7 | X-ray diffraction spectra of equal-capacity discharged electrodes (**a**) The X-ray diffraction (XRD) spectra of carbon paper (CP) electrodes after discharging the same capacity by the end discharge capacity of the LiTFSI test cell. Comparing the three cathode samples with narrowed 2-theta angle region, which are displayed on (**b, c**), the crystalline carbon peaks of the pristine carbon paper at 27 ^{o} and 55 ^{o} were diminished only with the electrode using the LiTFSI catholyte. This may be due to the early passivation of the carbon surface by lateral electrodeposition of Li_2S. The shoulder peak at the 2-theta angle of 26 ^{o}, which marked as the dot sign, can be ascribed to the deposition of insoluble lithium polysulfide (LiPS) according to the previous finding [2].

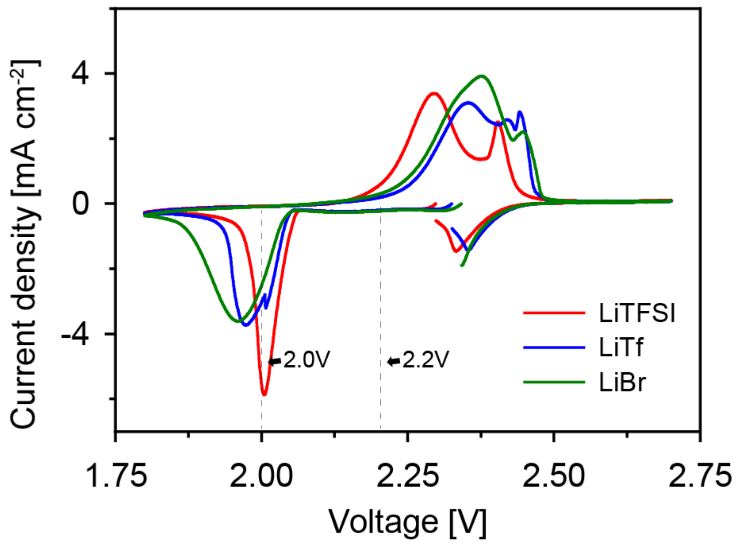

Supplementary Figure 8 | Cyclic Voltammograms of the salt varied lithium-sulfur cells with freestanding carbon nanotube electrodes Cyclic voltammetry (CV) (0.1 mV s^{-1}) of 1 M LiX (X= TFSI, Tf, or Br) based 0.2 M lithium polysulfide (LiPS) catholytes with carbon nanotube (CNT) freestanding cathodes and lithium metal anodes. Based on the position of the peak potentials from the cyclic voltammograms, 2.0 V is in the voltage range of 'kinetic-controlled' regime. The second discharge peak potentials from the CV diagrams are close to or lower than 2.0 V, indicating that the surface concentrations of the reactants have not depleted yet for all of the three cells, thus not 'diffusion-controlled' regime.

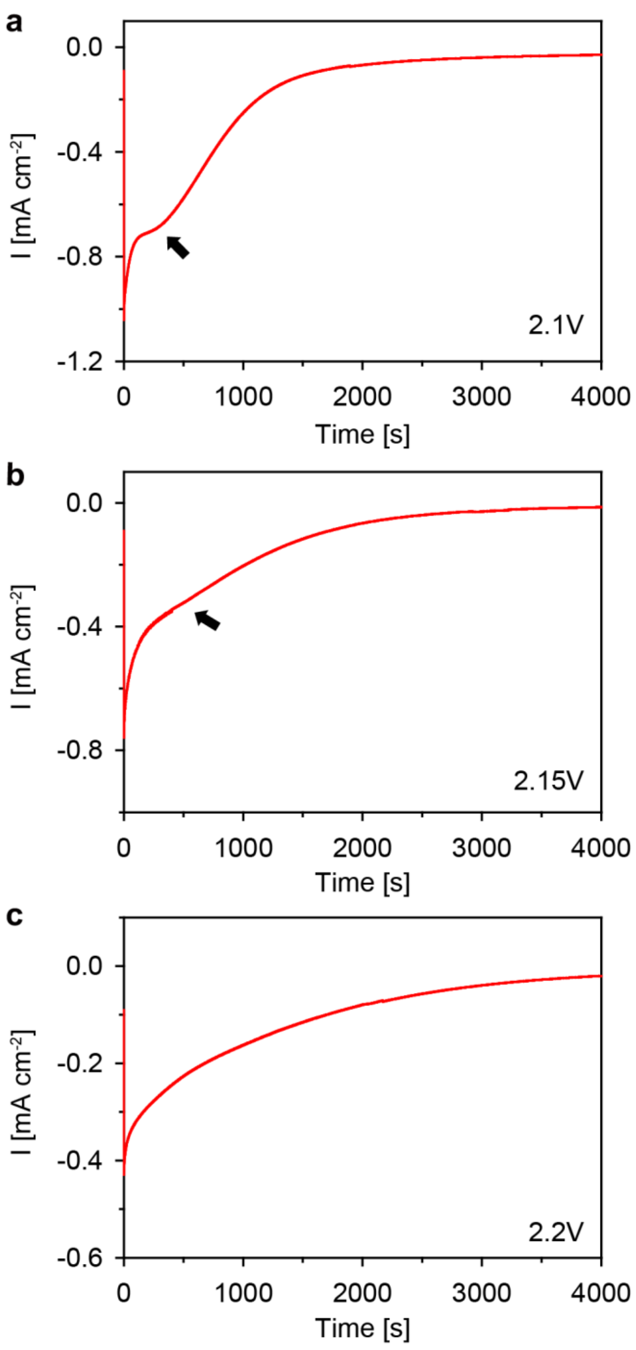

Supplementary Figure 9 | Current responses of potentiostatic discharges at different voltage ranges The current response data were recorded with different applied voltages of (**a**) 2.1 V, (**b**) 2.15 V, and (**c**) 2.2 V to confirm that 2.2 V is in an appropriate range for the pre-discharge process. As F. Y. Fan et al. noted in the previous work[3], the reduction of non-depositing sulfur species should show the exponentially decaying behavior, which follows the Avrami equation form (: $Y = 1\text{-}\exp(-Kt^n)$). If the potentiostatic discharge operation is with the process of electrodeposition of lithium sulfide (Li_2S), the current response does not follow the exponential decay function any longer. Based on the knowledge, at 2.1 V and 2.15 V, the Li_2S deposition reaction took place, while the response at 2.2 V discharge only showed a clear exponentially decaying behavior without Li_2S deposition.

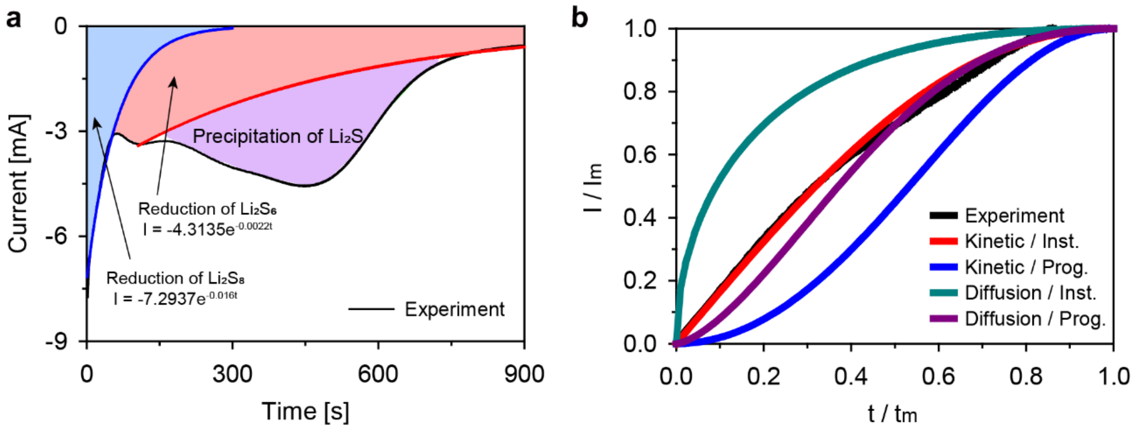

Supplementary Figure 10 | Fitting of the current response to theoretical two-dimensional nucleation and growth models. (**a**) Background fitting of current vs. time curve for Chronoamperometry (CA) test at 2.00 V (LiTFSI). The black curve (experiment data) was fitted as the sum of two exponential functions, assigned to the reduction of the residual Li_2S_8 and Li_2S_6 (blue and red, respectively), and a peak from the Li_2S electrodeposition[3]. (**b**) The extracted current response of lithium sulfide (Li_2S) electrodeposition (black) was compared to the four different nucleation and growth mathematical models[4,5]:

$$\text{Kinetic} - \text{limited/Instantaneous:}\quad \frac{I}{I_m} = \left(\frac{t}{t_m}\right) \exp\left[-\frac{1}{2}\left(\frac{t^2 - {t_m}^2}{{t_m}^2}\right)\right] \quad (S1)$$

$$\text{Kinetic} - \text{limited/Progressive:}\quad \frac{I}{I_m} = \left(\frac{t}{t_m}\right)^2 \exp\left[-\frac{2}{3}\left(\frac{t^3 - {t_m}^3}{{t_m}^3}\right)\right] \quad (S2)$$

$$\text{Diffusion} - \text{limited/Instant.:}\quad \left(\frac{I}{I_m}\right)^2 = \frac{1.9542}{t/t_m}\left[1 - \exp\left(-1.2564\frac{t}{t_m}\right)\right]^2 \quad (S3)$$

$$\text{Diffusion} - \text{limited/Progress.:}\quad \left(\frac{I}{I_m}\right)^2 = \frac{1.2254}{t/t_m}\left[1 - \exp\left(-2.3367\frac{t^2}{{t_m}^2}\right)\right]^2 \quad (S4)$$

(where I_m is the peak current, t_m is the corresponding time at which the I_m occurs.)

The current response fits to the kinetic-controlled/instantaneous nucleation and growth model based on I/I_m vs. t/t_m plots.

	LiTFSI	LiTf	LiBr
**t_m [s]**	345.41	547.56	835.20
**I_m [mA]**	-4.8808	-3.2191	-1.3522
**$N_0\ k_g^2$ [s^{-2}]**	1.82 x 10^{-9}	7.45 x 10^{-10}	3.30 x 10^{-10}

Supplementary Table. 1 | Calculation of the lateral growth rates of lithium sulfide upon different electrolytes The current vs. time responses at 2.0 V potentiostatic discharge, which were from electrodeposition of lithium sulfide (Li_2S), are interpreted with the Bewick, Fleischmann, and Thirsk (BFT) instantaneous theory model in Equation (S1) :

$$\frac{I}{I_m} = \left(\frac{t}{t_m}\right) \exp\left[-\frac{1}{2}\left(\frac{t^2 - {t_m}^2}{{t_m}^2}\right)\right] \qquad \text{(S1)}$$

I_m and t_m correspond to the current and time values when the maximum current flows, and can be expressed using parameters in Equation (S2) and (S3):

$$t_m = \left(\frac{\rho^2}{2\pi M^2 N_0 {k_g}^2}\right)^{\frac{1}{2}} \qquad \text{(S2)}$$

$$I_m = nFh(2\pi N_0 {k_g}^2)^{\frac{1}{2}} \exp\left(-\frac{1}{2}\right) \qquad \text{(S3)}$$

In the equations, N_0 represents the number density of nuclei and k_g is the lateral growth constant of a deposited material[6]. (M is the molecular weight, ρ is the density of deposits and h is the thickness of the layer.) Average values of t_m and I_m are provided from the five independent potentiostatic discharge experiments per each electrolyte. The lateral growth rates of Li_2S, $N_0\ k_g^2$ terms, are calculated by assigning the average t_m numbers in Equation (S2) for the electrolytes.

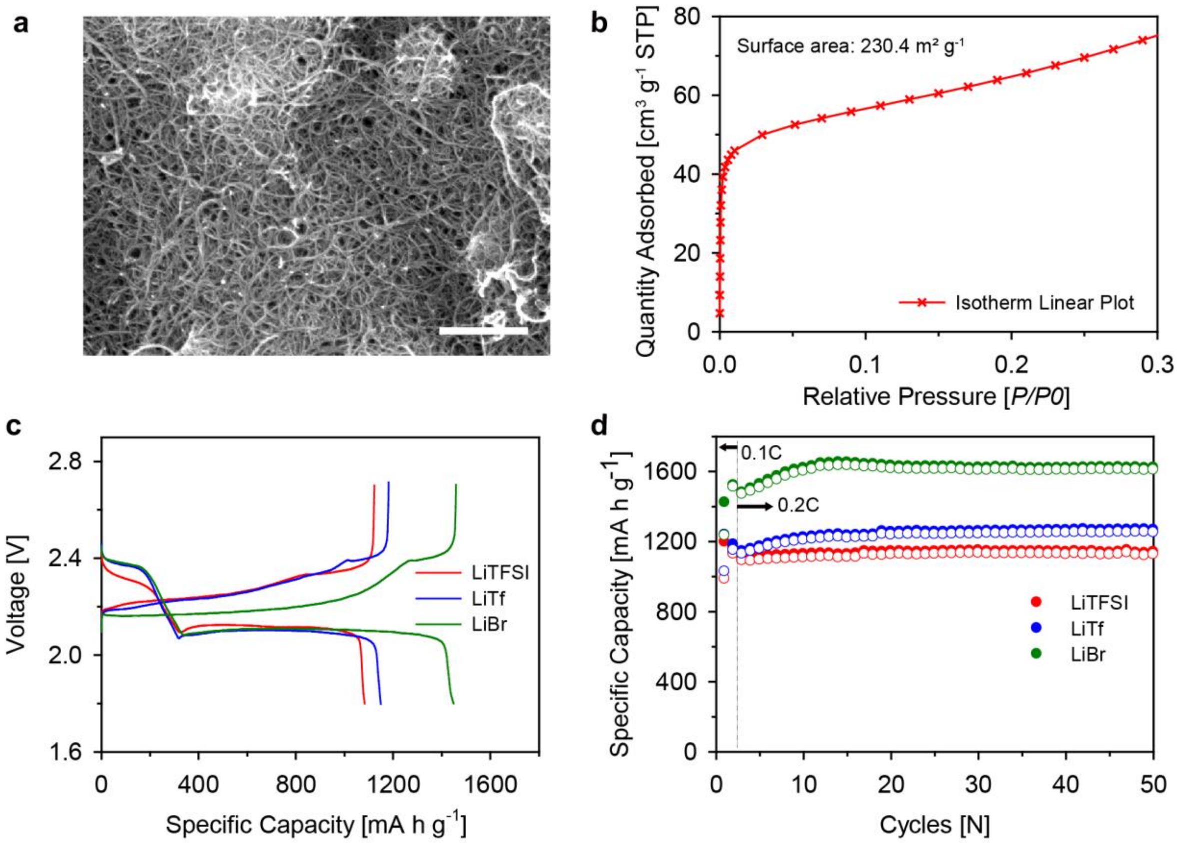

Supplementary Figure 11 | Electrochemical profiles of the three salt anions with the freestanding carbon nanotube electrode (**a**) A Scanning Electron Microscopy (SEM) image. The scale bar, 1 µm. (**b**) the Brunauer–Emmett–Teller (BET) isotherm curve of the carbon nanotube (CNT) freestanding electrode. The BET surface area of the CNT electrode was measured to be 230.4 m^2 g^{-1}. (**c**) Charge and discharge curves at the first cycle (0.2 C) and (**d**) the cycling stability at 0.2 C (closed circle: charge capacity, open circle: discharge capacity) for the CNT-based lithium-sulfur (Li-S) cells with the LiTFSI, LiTf, and LiBr electrolytes. The electrolytes consist of 0.2 M lithium polysulfide (LiPS, Li_2S_8 based) with 1 M Li salts (LiX, X= TFSI, Tf, or Br) / 0.2 M lithium nitrate ($LiNO_3$) / 1,3-dioxolane (DOL): 1,2-dimethoxyethane (DME) (1:1), Theoretical areal capacities: 1.68 mA h cm^{-2}

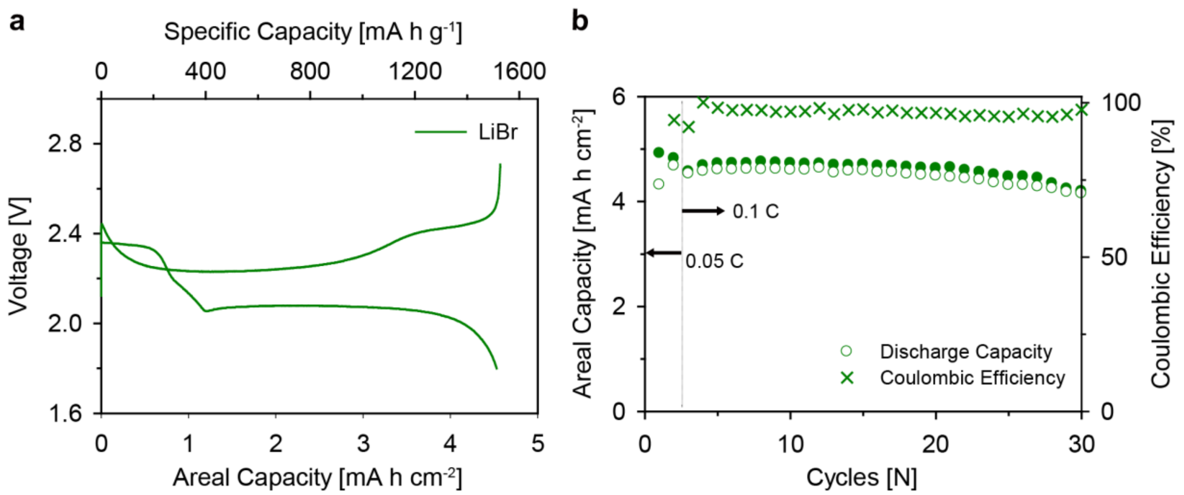

Supplementary Figure 12 | Electrochemical profiles of 3 mg cm^{-2} sulfur loaded lithium-sulfur cell with the LiBr electrolyte (**a**) Charge and discharge curves of the first 0.1 C cycle, (**b**) Cycling performance and Coulombic efficiency values for 30 charge/discharge cycles. 4.53 mA h cm^{-2} of areal capacity was achieved with the 3 mg cm^{-2} sulfur loading (Theoretical areal capacities: 5.03 mA h cm^{-2})

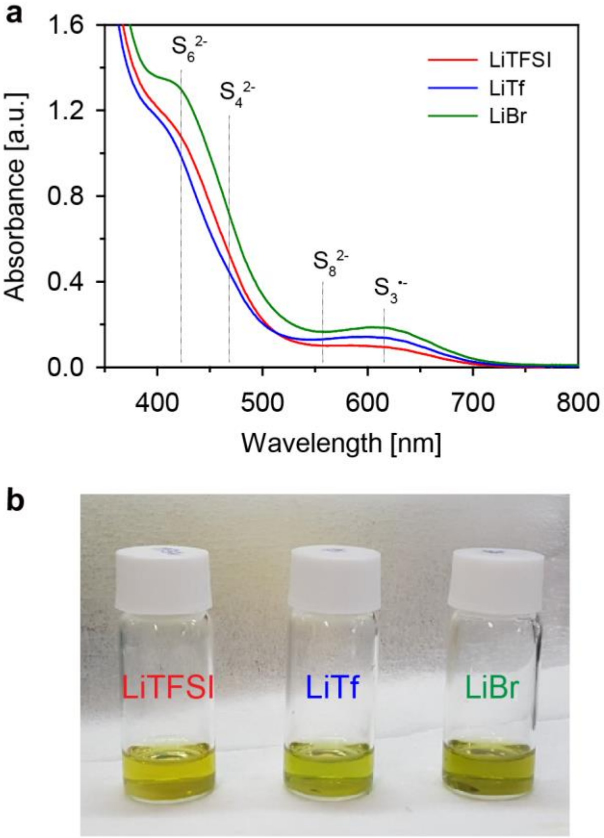

Supplementary Figure 13 | UV-Vis absorption spectra verifying distribution and amount of polysulfide anions 1 mM of lithium polysulfide (LiPS) solutions were prepared using the three salt anions to observe the polysulfide (PS) anion concentrations. (**a**) UV-Vis absorption spectra of LiPS solutions (Li_2S_8 based) with the three different supporting salts. (UV band attribution: S_8^{2-} at 560 nm, S_6^{2-} at 470 nm S_4^{2-} at 420 nm and $S_3^{\bullet-}$ at 617 nm[7]). It was previously reported that S_4^{2-} gives rise to a yellow color, and $S_3^{\bullet-}$ radical displays blue. (**b**) Comparing the LiTFSI sample to the LiTf and LiBr ones, when the donor number (DN) of the salt anion increases, the color turns to green, which indirectly supports the increasing population of $S_3^{\bullet-}$. Moreover, the LiBr electrolyte shows a deeper color gradient, expecting the higher concentrations of the all solvated PS anions. Two figures show the correspondences in polysulfide anions' distribution and their amounts in the electrolytes.

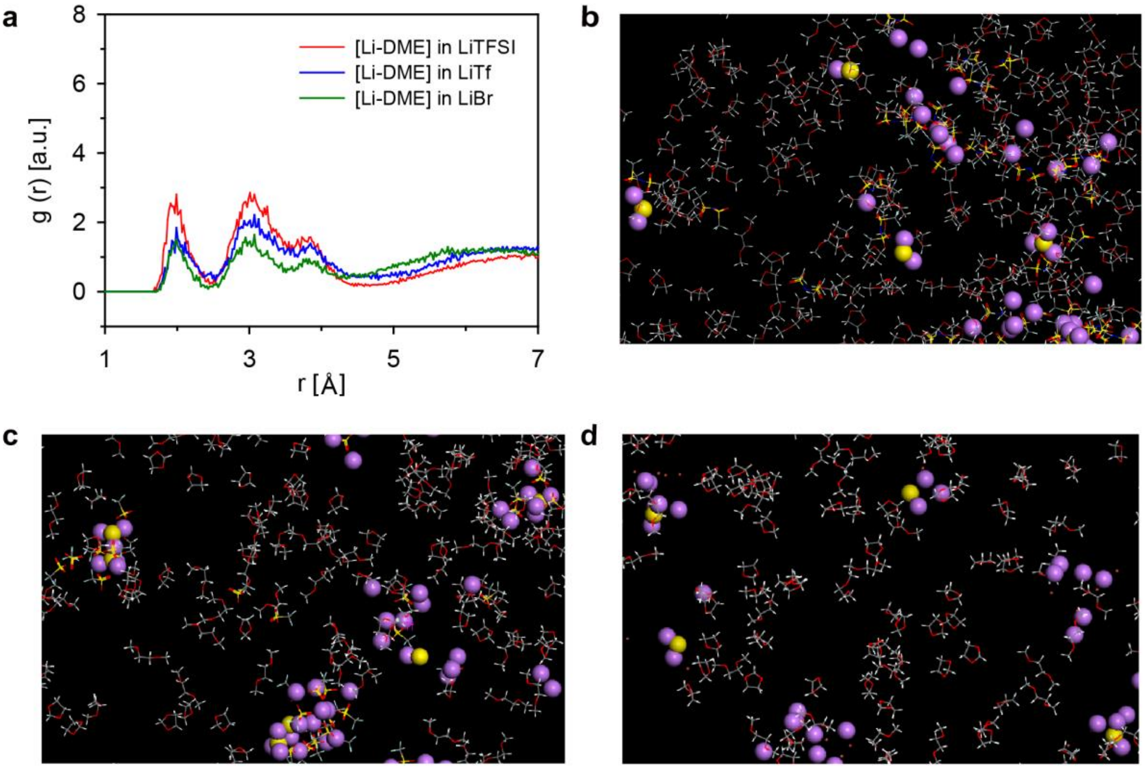

Supplementary Figure 14 | Molecular Dynamics simulation in different salt systems (**a**) Radial distribution functions (RDF) of 1,2-dimethoxyethane (DME) molecules from lithium ion (Li^+) under 1 M LiX (X= TFSI, Tf, or Br) in 1,3-dioxolane (DOL): 1,2-dimethoxyethane (DME) (1:1) solutions. As increasing the anion's donor number (DN), the solvent molecules less participate in the solvation cluster of Li^+ due to stronger affinity of anions with Li^+. Enlarged snapshots of the molecular dynamics (MD) simulation cells of (**b**) LiTFSI, (**c**) LiTf, and (**d**) LiBr salt containing electrolytes with sulfide (S^{2-}) anions. The environments adjacent to S^{2-} anions were observed. (Purple: Li^+, Yellow: S^{2-})

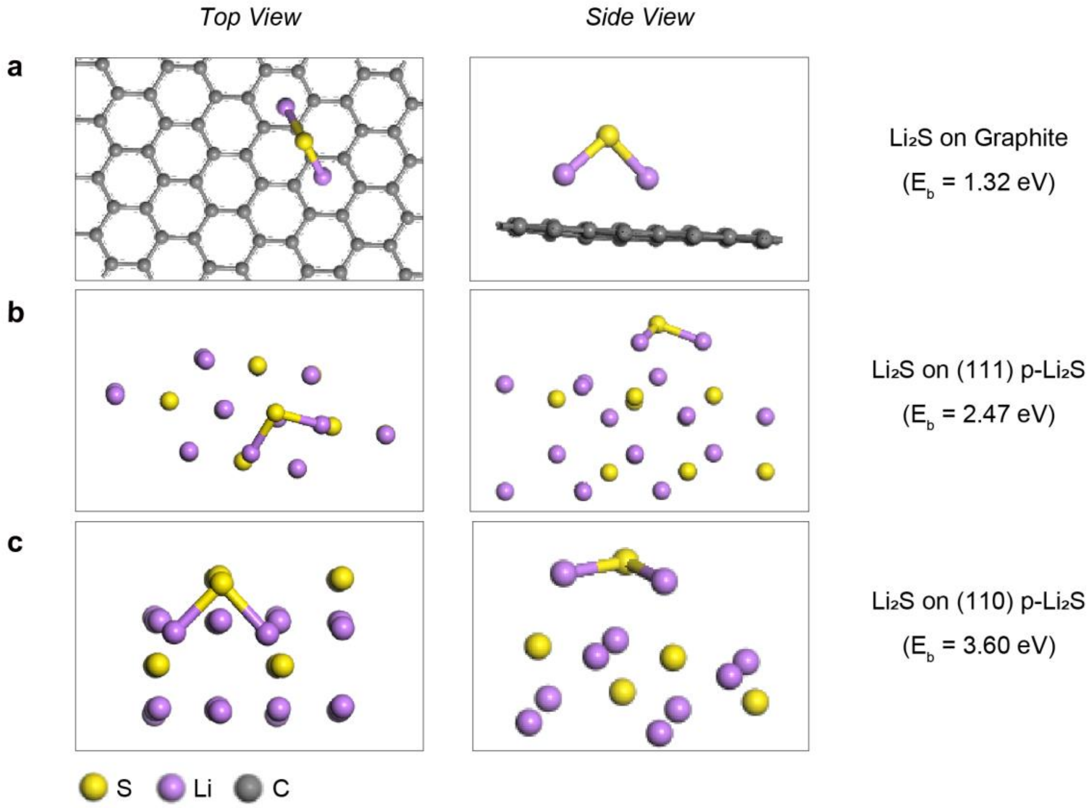

Supplementary Figure 15 | Binding energy calculation of a lithium sulfide molecule on different interfaces The binding energy values of a lithium sulfide (Li_2S) molecule on (**a**) graphite, (**b**) (110) plane, and (**c**) (111) plane of Li_2S precipitate (p-Li_2S) interfaces. Two specific facets of p-Li_2S, (110) and (111), were selected based on the peaks matched to the X-ray Diffraction (XRD) spectra library of Li_2S (MDI Jade 5.0, PDF#04-0836)[8]. The binding energy (E_b) was computed by the equation as following: $E_b = E_{Li2S} + E_{surface} - E_{Li2S+surface}$, where $E_{Li2S+surface}$ is the system energy of which a Li_2S molecule adsorbed on the surface; E_{Li2S} and $E_{surface}$ represents the energy of an unbound Li_2S molecule and that of a pristine surface, respectively. According to the equation, the higher positive value of E_b indicates the stronger interaction between the newly produced Li_2S and the surface substrate.

References for Supplementary Information

1 Dobson, J. C., McLarnon, F. R. & Cairns, E. J. The corrosion of some metals in sulfur-polysulfide melts. *Corrosion Science* **28**, 953-967, doi:https://doi.org/10.1016/0010-938X(88)90014-5 (1988).

2 Conder, J. *et al.* Direct observation of lithium polysulfides in lithium–sulfur batteries using operando X-ray diffraction. *Nature Energy* **2**, 17069, doi:10.1038/nenergy.2017.69 https://www.nature.com/articles/nenergy201769#supplementary-information (2017).

3 Fan Frank, Y., Carter, W. C. & Chiang, Y. M. Mechanism and Kinetics of Li2S Precipitation in Lithium–Sulfur Batteries. *Advanced Materials* **27**, 5203-5209, doi:10.1002/adma.201501559 (2015).

4 Bewick, A., Fleischmann, M. & Thirsk, H. R. Kinetics of the electrocrystallization of thin films of calomel. *Transactions of the Faraday Society* **58**, 2200-2216, doi:10.1039/TF9625802200 (1962).

5 Scharifker, B. & Hills, G. Theoretical and experimental studies of multiple nucleation. *Electrochimica Acta* **28**, 879-889, doi:https://doi.org/10.1016/0013-4686(83)85163-9 (1983).

6 Jafarian, M., G. Mahjani, M., Gobal, F. & Danaee, I. *Effect of potential on the early stage of nucleation and growth during aluminum electrocrystallization from molten salt (AlCl 3–NaCl–KCl)*. Vol. 588 (2006).

7 Zou, Q. & Lu, Y.-C. Solvent-Dictated Lithium Sulfur Redox Reactions: An Operando UV–vis Spectroscopic Study. *The Journal of Physical Chemistry Letters* **7**, 1518-1525, doi:10.1021/acs.jpclett.6b00228 (2016).

8 Liu, Z., Hubble, D., Balbuena, P. B. & Mukherjee, P. P. Adsorption of insoluble polysulfides Li2Sx (x = 1, 2) on Li2S surfaces. *Physical Chemistry Chemical Physics* **17**, 9032-9039, doi:10.1039/C4CP06118G (2015).
